# School-based participatory health education for malaria control in Ghana: engaging children as health messengers

**DOI:** 10.1186/1475-2875-9-98

**Published:** 2010-04-18

**Authors:** Irene Ayi, Daisuke Nonaka, Josiah K Adjovu, Shigeki Hanafusa, Masamine Jimba, Kwabena M Bosompem, Tetsuya Mizoue, Tsutomu Takeuchi, Daniel A Boakye, Jun Kobayashi

**Affiliations:** 1 West African Centre for International Parasite Control, Parasitology Department, Noguchi Memorial Institute for Medical Research, University of Ghana, Legon, Accra, Ghana; 2 Department of Community and Global Health, Graduate School of Medicine, the University of Tokyo, 7-3-1 Hongo, Bunkyo, Tokyo, Japan; 3 Department of Epidemiology and International Health, International Clinical Research Center, National Center for Global Health and Medicine, 1-21-1 Toyama, Shinjuku, Tokyo, Japan; 4 Zoology Department, University of Ghana, Legon, Accra, Ghana; 5 Bureau of International Medical Cooperation, National Center for Global Health and Medicine, 1-21-1 Toyama, Shinjuku, Tokyo, Japan; 6 Department of Tropical Medicine and Parasitology, School of Medicine, Keio University, 35 Shinano-machi, Shinjuku, Tokyo, Japan

## Abstract

**Background:**

School children have been increasingly recognized as health messengers for malaria control. However, little evidence is available. The objective of this study was to determine the impact of school-based malaria education intervention on school children and community adults.

**Methods:**

This study was conducted in the Dangme-East district of the Greater Accra Region, Ghana, between 2007 and 2008. Trained schoolteachers designed participatory health education activities and led school children to disseminate messages related to malaria control to their communities. Three schools and their respective communities were chosen for the study and assigned to an intervention group (one school) and a control group (two schools). Questionnaire-based interviews and parasitological surveys were conducted before and after the intervention, with the intervention group (105 children, 250 community adults) and the control group (81 children, 133 community adults). Chi-square and Fisher's Exact tests were used to analyse differences in knowledge, practices, and parasite prevalence between pre- and post-intervention.

**Results:**

After the intervention, the misperception that malaria has multiple causes was significantly improved, both among children and community adults. Moreover, the community adults who treated a bed net with insecticide in the past six months, increased from 21.5% to 50.0% (*p *< 0.001). Parasite prevalence in school children decreased from 30.9% to 10.3% (*p *= 0.003). These positive changes were observed only in the intervention group.

**Conclusions:**

This study suggests that the participatory health education intervention contributed to the decreased malaria prevalence among children. It had a positive impact not only on school children, but also on community adults, through the improvement of knowledge and practices. This strategy can be applied as a complementary approach to existing malaria control strategies in West African countries where school health management systems have been strengthened.

## Background

Ghana is located in sub-Saharan Africa, where an estimated 90% of the world's malaria-attributable deaths occur. In Ghana, malaria accounts for more than 44% of reported outpatient visits and an estimated 22% of deaths in children under the age of five. Reported malaria cases represent only a small proportion of the actual number of episodes, as the majority of people with symptomatic infections are treated at home and are, therefore, not often reported [1,2]. In Ghana, the national malaria control programme focuses chiefly on pregnant women and children under five years of age, as malaria leads to more serious consequences in this group. The main activities of the malaria control programme in Ghana are facility-based and implemented at the health centre level, i.e., providing intermittent preventive treatment (IPT) and distributing insecticide-treated bed nets (ITNs) to pregnant women during antenatal care, and distributing ITNs to women with young children during immunization [3].

Children of school-going age have also been targeted for malaria control in some endemic countries including Ghana [4-6]. As more and more children attend school, governments are increasingly recognizing the importance of child health for educational achievement [7]. A number of studies that focus primarily on evaluating the effectiveness of providing treatment for children have been conducted in the school setting, [5,8,9]. Recently, the impact of IPT among school children has drawn increased attention [10-12]. However, while IPT is relevant only in high-transmission areas, skills-based health education for malaria control is recommended to be effective in all transmission settings [13].

Recently, school-based health education interventions have been conducted for malaria control. They use an innovative approach that engages school children to reach community adults with health messages and hygienic practices through action-oriented and participatory learning action (PLA). For example, Okabayashi *et al *[14] reported that school children disseminated information on malaria to the community through a variety of approaches including issuing newsletters, placing billboards, and holding village events in Thailand. As a result, both children and community adults showed improved knowledge, attitudes, and practices pertaining to malaria. Onyango-Ouma *et al *[15] evaluated the potential of school children as health change agents in a rural community in Kenya and observed improved knowledge pertaining to malaria among children and guardians. In Lao PDR, Nonaka *et al *[16] demonstrated that school children reached out to and influenced women who were not caregivers of target school children to improve their behaviour in relation to malaria control. These studies consistently reported that school children were not merely recipients of health education, but also contributed to malaria control by playing the role of health change agents in the community. In the present study, this approach was employed in a different area in order to confirm the effectiveness of the strategy, with the consideration that socio-cultural factors in the different area might influence its effectiveness.

The West African Centre for International Parasite Control (WACIPAC) was established at the Noguchi Memorial Institute for Medical Research of the University of Ghana in 2004, with support from the Japan International Cooperation Agency. As part of the strategy to control parasitic diseases in the West African sub-region of sub-Saharan Africa, the WACIPAC set up a parasite control project model site in the Ada-Foah sub-district of the Dangme-East District to facilitate capacity building and evaluate intervention strategies. In this study, the objective was to determine the impact of school-based malaria education intervention, not only on children but also on adult community members.

## Methods

### Study site

The study was conducted in the Dangme-East District of the Greater Accra Region, Ghana, between July 2007 and June 2008. Dangme-East District is located approximately 100 km east of the capital, Accra. The district is partially rural and partially urban in nature and covers an area of 721 km^2^. The indigenous Ga-Dangme people form the majority of the residents in the area. According to the nation's 2000 census, the population of the district is 93,193. Annual rainfall ranges between 740 and 900 mm. The highest and lowest mean monthly temperatures are approximately 30°C and 26°C, respectively. The relative humidity throughout the year ranges between 65 and 80%. The region's vegetation is basically coastal savannah, consisting of grass with isolated patches of trees and shrubs. There are wet and dry seasons; the wet season begins in May and ends in October. The district has four government-run health centres and a district hospital. According to the data available at the District Health Office, in 2006, the malaria prevalence of clinically diagnosed and laboratory-confirmed cases among outpatients in the district was 45.0% (19,273/42,974).

**Figure 1:**
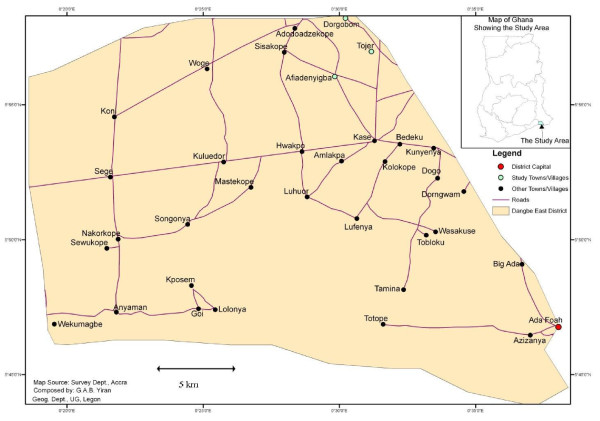
**Map of the Dangme-East District showing the study villages**. Small map inserted at the upper right shows Ghana; Dangme-East District is highlighted in green. Green circles indicate the locations of the study villages and schools (Afiadenyigba, Dorgobom and Tojeh).

In collaboration with local education and health authorities, three primary schools (Afiadenyigba, Dorgobom and Tojeh) were purposively selected from the 15 located within the Kasseh-East Circuit of the Dangme-East District Education Directorate of the Ghana Education Service. The selection was based on specified criteria including: similar ecological setting, school master's willingness to participate in the study, and school master's recognition of malaria as one of the major problems affecting school children. The selected schools are located within several kilometres of each other, in rural settings (Figure 1). The Afiadenyigba village and school served as the intervention area whilst the Dorgobom and Tojeh villages and schools were used as controls. According to the sample size calculation, approximately one hundred children were required in each of the intervention and control groups. As neither the Dorgobom nor Tojeh schools had enough children to reach the required sample size, the two control schools and their respective communities were grouped as one.

### Study populations

The study population consisted of 186 children in grades 3-5 from the selected schools and 383 adults who resided in villages where the schools are located. School children in the first and second grades were excluded due to perceived difficulties in involving them in the intervention. For community adults, an attempt was made to invite one adult participant from each household of the villages where the schools are located. In recruitment of adult participants, priority was given to those that were caregivers of children at the target school; in case there were no household members who were caregivers of target school children, priority was given to caregivers of non-target children or any woman of reproductive age.

### Intervention

#### Teacher training

The health education intervention started from October 2007. At the beginning of the intervention, a two-day training was conducted for all teachers at the intervention school. On the first day, malaria-related information was shared, such as mosquito biology, malaria signs and symptoms, and treatment and prevention. Additionally, teaching methods using PLA were introduced. On the second day, strategies for effective implementation of malaria education activities in the schools and villages were discussed. Finally, teachers developed action plans for the health education intervention (See Additional file 1).

#### Post-teacher training activities

The research team (IA, DN, JKA, and SH) provided picture charts and posters on malaria transmission and prevention, which were requested by teachers and used in teaching children in the participating grades. The posters were displayed on the walls of their classroom after teaching. Teachers guided the children through dramatizing the transmission of malaria and prevention methods. The teachers then led the children to observe their school compound and cleared possible mosquito breeding sites such as open cans and dumped containers. The children were also encouraged to draw pictures on malaria according to their understanding of the malaria education they received and voluntarily use the pictures to educate their peers and adults in the village. The teachers composed a song in the local language (Ga-Dangme) on malaria entitled 'pumi (mosquito) song', to educate the children and community on malaria transmission and prevention. The song, which became popular, was aimed at correcting common misperceptions that eating green mangoes or standing in the sun gives malaria and it promoted the use of bed nets for preventing mosquito bites and malaria transmission. The teachers made slogans such as "mosquito: malaria provider" which the children happily chanted at school and in the village. These activities were mainly conducted in the morning before the day's lessons.

#### Campaign

In February 2008, the teachers and 3^rd ^to 5^th ^grade children of the intervention school also conducted a one-day anti-malaria campaign in which they educated the village residents on malaria through a number of recreational activities. First, they marched through the village singing the 'pumi song' accompanied by the school band, at which time they invited community members to their school compound for a durbar. At the gathering, the children educated the people about malaria through drama and poetry recitals. A community health nurse demonstrated the correct procedure for treatment of conventional bed nets using insecticide tablets and explained the benefits of sleeping under treated bed nets and other malaria information. Volunteer residents had their conventional bed nets freshly treated or re-treated at no cost.

### Surveys

#### Questionnaire-based interview

Pre- and post-intervention questionnaire-based interviews on malaria-related knowledge and practices were conducted with all study participants (186 children and 383 adults) from July to September, 2007, and April to June, 2008, respectively. Questionnaires were originally developed on the basis of previous studies investigating perceptions, knowledge and behaviours pertaining to malaria in Ghana and other countries [17-20]. The questionnaires were modified appropriately after pre-test was conducted with 22 mothers and 38 school children. There were two questionnaires: one for school children and one for community adults. Each of the questionnaires shared 14 question items pertaining to knowledge and five pertaining to practices. The questionnaire for adults contained three additional items pertaining to practices. For the post-intervention questionnaire interviews, three more question items were included to verify whether the community adults were exposed to the malaria education undertaken by the children. Native speakers of the Ga-Dangme language administered the questionnaire-based interviews. Before the administration of questionnaire, the interviewers were trained for a day by one of the authors (JKA) who is a native speaker of Ga-Dangme. Each of the participating children was interviewed at school. The adult participants were interviewed at home.

#### Observational survey

The research team made an observational survey on conditions in and around the households before the intervention in August, 2007. The observation with a simple check-list focused on possible mosquito breeding sites around the house, presence or absence of mosquito-proof netting on windows/trap doors and bed nets, and the condition of those mosquito prevention tools. As entering the house was perceived as an intrusion of privacy, the researchers asked an adult member of the households to bring one net, even though some of the households had multiple nets.

#### Parasitological survey

Finger-prick blood for preparation of thick and thin films on microscope glass slides was taken from the school children that volunteered in the intervention and control schools. The blood films were Giemsa-stained after fixing the thin film in methanol; they were then examined microscopically to detect malaria parasites. This exercise was performed pre-intervention in October 2007 and post-intervention in June 2008.

### Data analysis

The sample size of 91 children in each group was calculated to detect statistical significance in differences in prevalence of malaria between pre- and post-intervention, with 80% power at 95% significance level. It was hypothesized that the initial prevalence would be 40%, and that a 20% decrease would be achieved in the intervention school.

Baseline differences in socio-demographic variables, conditions of malaria prevention tools, and possible mosquito breeding site were analysed by Chi-square test or Mann-Whitney U test. Differences between pre- and post-intervention in knowledge, practices, and parasite prevalence were analysed by Chi-square test or Fisher's Exact test. Statistical analysis was performed with SPSS 17.0 (SPSS Inc., Chicago, IL). A *P *value of < 0.05 was accepted as statistically significant.

### Ethical clearance

Approval and ethical clearance for the study were obtained from the Scientific and Technical Committee and the Institutional Review Board, respectively, of the Noguchi Memorial Institute for Medical Research with the certified protocol number: CPN 038/06-07. Participants were informed that their participation was purely voluntary and assured of the confidentiality of all data collected. Informed written consent was obtained from adult participants, and parent/guardian of school children, with the children giving their assent. Verbal consent was obtained from participating children before conducting the survey.

## Results

### Study participants

From the total of 217 school children in the target grades, 186 participated both in the pre-and post-intervention questionnaire-based interviews and were used for analysis. Participants consisted of 105 children (59 boys) out of a total of 128 in the intervention school, and 81 children (44 boys) out of a total of 89 in the control schools (Table 1). The median age was 13.0 (range: 8-20) years in the intervention school, and 12.0 (range: 8-21) in the control schools. No baseline difference was found between the intervention and control school children in age, sex, or distribution ratio by grade.

**Table 1:** Characteristics of school children.

Characteristic	Intervention school (n = 105)	Control schools (n = 81)	*p*-value^a^
Age (yrs), median (range)	13 (8--20)	12 (8-21)	0.132^b^
Sex, male/female	59/46	44/37	0.799
Grade: n (%)			0.598
3^rd^	35 (33.3)	31 (38.3)	
4^th^	37 (35.2)	23 (28.4)	
5^th^	33 (31.4)	27 (33.3)	

^a^, Chi-square test

^b^, Mann-Whitney U test

Of a total of 447 households, 383 were involved in the study and one participant from each household participated in the questionnaire-based interviews. Of the adult participants, 115 were caregivers of target children. Adult participants consisted of 250 adults (241 women) from the intervention village and 133 (125 women) from the control villages (Table 2). Median age was 35.0 (range: 16 to 93) years in the intervention village, and 35.5 (range: 18 to 85) in the control villages. Most of the adults were engaged in farming (intervention village 68.0%, control villages 77.4%); over 90% were native Ga-Dangme speakers. Most of them had elementary or no formal education.

**Table 2:** Characteristics of the community adults.

Characteristic	Community Adults		
	
	Intervention village (n = 250)	Control villages (n = 133)	*p*-value^a^
Age(yrs), median (range)	35.0 (16-93)	35.5 (18-85)	0.504^b^
Sex, male/female	9/241	8/125	0.275
Occupation, n (%)			0.146
Farmer	170 (68.0)	103 (77.4)	
Trader	43 (17.2)	17 (12.8)	
Others	37 (14.8)	13 (9.8)	
Education, n (%)			0.162
No formal education	86 (34.4)	56 (42.1)	
Elementary (primary or middle)	120 (48.0)	63 (47.4)	
Senior (secondary or higher)	16 (6.4)	3 (2.3)	
Other	28 (11.2)	11 (8.2)	
Ethnicity: n (%)			0.271
Ga-Dangme	235 (94.0)	121 (91.0)	
Other	15 (6.0)	12 (9.0)	

^a^, Chi-square test

^b^, Mann-Whitney U test

### Questionnaire-based interview

According to the post-intervention interview, 23.1% (24/104) of the children in the intervention school responded that they had, at least once, presented a picture to family members and/or other community members. For adults in the intervention community, 37.1% (92/248) confirmed that a child showed a picture to them at least once; 80.7% (201/249) responded they had heard the "pumi song" sung by the school children; and 59.0% (147/249) responded that they attended the durbar at the intervention school.

School children in the intervention school significantly improved their knowledge on ITN, cause of malaria, mosquitoes, and paracetamol. Before the intervention, poor knowledge on cause of malaria according to response to question item was striking. After the intervention, those who correctly responded to the question items "Mango cannot cause malaria" increased from 10.5% to 79.8% (*p *< 0.001), from 11.4% to 75.0% on "Heat from the sun cannot cause malaria" (*p *< 0.001), and from 16.3% to 43.8% on "Drinking dirty water cannot cause malaria" (*p *< 0.001) (Table 3). Among school children in the control schools, the difference between pre- and post- intervention was only statistically significant in one item. In the practice questions, no positive change was found in the intervention group of children. Rather, "Talking with neighbours about malaria" and "Burning something to prevent malaria" decreased with statistical significance (*p *= 0.043, *p *= 0.012). In control school children, "Talking with neighbours about malaria" and "Covering arms and legs when going outside at night" also showed a statistically significant decrease (*p *= 0.002, *p *< 0.001).

**Table 3:** Comparison of changes in knowledge and practices among school children.

Knowledge/Practices	Knowledge: % of respondents who knew Practices: % of respondents who practiced very often or sometimes					
	
	Intervention (n = 105)			Control (n = 81)		
	
	Before (%)	After (%)	*p*-value^a^	Before (%)	After (%)	*p*-value^a^
Knowledge						
Meaning of ITN	80.4	90.5	0.039	88.9	81.5	0.185
Effectiveness of ITN against mosquitoes	96.3	95.8	1.000^b^	98.6	91.0	0.054^b^
Necessity of ITN re-treatment	30.5	55.2	0.001	36.1	37.9	0.830
Vulnerability of ITN to sunshine exposure	46.8	58.7	0.121	38.9	42.4	0.673
Place to get re-treatment service for ITN	69.5	92.6	<0.001	66.7	72.7	0.440
Paracetamol alone cannot cure malaria	34.0	69.5	<0.001	46.9	55.0	0.305
Breeding site of mosquitoes	42.2	74.3	<0.001	32.1	53.1	0.007
Habitat of mosquito larvae	94.3	95.2	1.000^b^	95.1	92.6	0.746^b^
Resting place for mosquitoes	91.4	93.3	0.603	93.8	95.1	1.000^b^
Mango cannot cause malaria	10.5	79.8	<0.001	18.5	4.9	0.013^b^
Heat from the sun cannot cause malaria	11.4	75.0	<0.001	6.3	6.3	1.000^b^
Mosquito bites can cause malaria	76.2	99.0	<0.001	90.1	90.1	1.000
Drinking dirty water cannot cause malaria	16.3	43.8	<0.001	8.6	8.6	1.000
Sex difference in biting behaviour of mosquitoes	15.2	24.8	0.084	5.0	5.0	1.000^b^
Practice						
Weeding around house in the past 6 months	90.5	90.5	1.000	85.2	80.2	0.406
Talking with family members about malaria in the past 6 months	55.2	43.3	0.084	17.5	7.6	0.060
Talking with neighbours about malaria in the past 6 months	51.4	37.5	0.043	22.2	4.9	0.002^b^
Burning something to prevent mosquitoes in the past 6 months	92.4	80.4	0.012	88.9	78.8	0.080
Covering arms and legs when going outside at night in the past 6 months	79.0	70.2	0.141	82.3	55.6	<0.001

^a^, Chi-square test

^b^, Fisher's Exact test

As shown in children, adult participants' knowledge on ITN, cause of malaria, mosquitoes, and paracetamol also significantly changed in the intervention area. In most of the question items, the percentage of respondents who had correct knowledge was slightly higher in adult participants than in children before the intervention. However, adult participants who believed that mango, heat from the sun, or drinking dirty water can cause malaria accounted for more than 80%. This knowledge was significantly improved after the intervention (Table 4). In contrast, adult participants in the control area did not show an increase in any knowledge question items. In the practice section, the percentage of those who treated a bed net with insecticide in the past six months significantly increased from 21.5% to 50.0% in the intervention area (*p *< 0.001), although it was almost the same between pre- (25.3%) and post-intervention surveys (30.5%) in the control area. In the intervention area, the percentages significantly decreased in "Sleeping under bed net" (*p *= 0.006), "Weeding around house" (*p *< 0.001), "Talking with children about malaria (*p *< 0.001), and "Talking with neighbours about malaria" (*p *< 0.001). In the control area, the percentages also significantly decreased in "Weeding around house" (*p *= 0.003) and "Covering arms and legs when going outside at night" (*p *= 0.013).

**Table 4:** Comparison of changes in knowledge and practices among community adults.

Knowledge/Practices	Knowledge: % of respondents who knew Practices: % of respondents who practiced very often or sometimes					
	
	Intervention (n = 250)			Control (n = 133)		
	
	Before (%)	After (%)	*p*-value^a^	Before (%)	After (%)	*p*-value^a^
Knowledge						
Meaning of ITN	86.3	74.6	0.006	93.1	89.1	0.266
Effectiveness of ITN against mosquitoes	84.7	93.2	0.006	86.9	84.7	0.635
Necessity of ITN re-treatment	51.4	52.1	0.891	59.5	33.6	<0.001
Vulnerability of ITN to sunshine exposure	54.9	75.1	<0.001	65.0	64.1	0.885
Place to get re-treatment service for ITN	62.5	72.4	0.032	63.6	65.0	0.832
Paracetamol alone cannot cure malaria	66.0	74.3	0.044	73.8	63.2	0.062
Breeding site of mosquitoes	57.4	66.7	0.034	65.4	51.1	0.018
Habitat of mosquito larvae	96.8	94.4	0.184	95.5	91.7	0.210
Resting place for mosquitoes	96.4	95.2	0.492	99.2	97.7	0.622^b^
Mango cannot cause malaria	15.6	61.1	<0.001	11.6	10.5	0.776
Heat from the sun cannot cause malaria	12.4	47.1	<0.001	4.6	2.3	0.334^b^
Mosquito bite can cause malaria	95.2	92.2	0.171	97.0	97.7	1.000^b^
Drinking dirty water cannot cause malaria	16.9	40.9	<0.001	3.0	2.3	1.000^b^
Sex difference in biting behaviour of mosquitoes	8.4	30.5	<0.001	0.8	3.0	0.370^b^
Practices						
Sleeping under bed net in the past 6 months	99.0	93.6	0.006^b^	92.9	94.9	0.525
Washing bed net in the past 6 months	95.7	93.6	0.391	91.3	95.3	0.280^b^
Treating bed net with insecticide in the past 6 months	21.5	50.0	<0.001	25.3	30.5	0.406
Weeding around house in the past 6 months	94.8	85.0	<0.001	94.7	83.1	0.003
Talking with children about malaria in the past 6 months	73.0	49.4	<0.001	40.6	34.8	0.334
Talking with neighbours about malaria in the past 6 months	43.4	33.1	<0.001	27.8	24.2	0.507
Burning something to prevent mosquitoes in the past 6 months	83.9	79.5	0.212	71.8	78.2	0.227
Covering arms and legs when going outside at night in the past 6 months	79.0	80.3	0.721	65.9	50.8	0.013

^a^, Chi-square test

^b^, Fisher's Exact test

### Observational survey

One hundred and seventy-two out of 263 (65.4%) households in the intervention area and 104 out of 184 (56.4%) households in the control areas were observed. Most of the households (intervention area: 86.6%, control area: 82.7%) possessed at least one bed net (Table 5). Among the nets observed, nearly half (46.3%, 43.0%) were conventional nets. Most of the nets (76.5%, 70.9%) had no obvious holes or tears through which mosquitoes could invade. In nearly half of the households (48.3%, 47.1%), a dumped container, which could be a potential breeding site for mosquitoes, was found in the compound. Baseline differences were observed in the condition of mosquito proof nets and covers for water storage; fewer mosquito proof nets were furnished on the windows in intervention households (20.9%) than in control households (44.2%) (*p *< 0.001). In contrast, more mosquito proof nets were intact in the intervention households (69.4%) than in control households (41.3%) (*p *= 0.011). Water storages were more likely to be covered in the intervention households (77.9%) than in control households (67.3%) (*p *= 0.028).

**Table 5:** Results of household observational survey before the intervention.

Item/condition observed	Community households		
	
	Intervention (n = 172)	Control (n = 104)	*p*-value^a^
Bed nets			
Possession of at least one net	149 (86.6%)	86 (82.7%)	0.558
Type of nets			
Long-lasting insecticide-treated nets	80 (53.7%)	49 (57.0%)	0.626
Conventional nets	69 (46.3%)	37 (43.0%)	
State of the nets			
Intact	114 (76.5%)	61 (70.9%)	0.618
Torn/hole	29 (19.5%)	21 (24.4%)	
Not in use	6 (4.0%)	4 (4.7%)	
Mosquito-proof nets on windows			
Furnished	36 (20.9%)	46 (44.2%)	<0.001
State of the nets			
Intact	25 (69.4%)	19 (41.3%)	0.011
Torn/hole	11 (30.6%)	27 (58.7%)	
Household compound			
Presence of trash container that can be breeding site of mosquitoes	83 (48.3%)	49 (47.1%)	0.722
Presence of covered water storage facility	134 (77.9%)	70 (67.3%)	0.028

^a^, Chi-square test

### Parasitological survey

Of the 105 children at the intervention school who participated in the questionnaire survey, 64.8% (68/105) participated in both pre- and post-intervention surveys; at the control schools, 77.8% (63/81) participated. In the intervention school, the median age (range) and male:female ratio were 14 (9-20) and 40:28, respectively, in the participating children; and 14 (8-18) and 19:18, respectively, in the non-participating children. In the control schools, the median age (range) and male:female ratio was 14 (10-19) and 29:34, respectively, in the participating children; and 15 (11-21) and 15:3, respectively, in the non-participating children. The difference in the male:female ratio between participating and non-participating children was statistically significant in the control schools (*p *= 0.007). *Plasmodium falciparum *was the only malaria parasite found among the school children. Parasite prevalence significantly reduced from 30.9% to 10.3% (*p *= 0.003) at post-intervention in the intervention school, while it increased from 9.5% to 15.9% in the control schools (Table 6).

**Table 6:** Prevalence of *P. falciparum *infection among target school children before and after intervention.

School children	*P. falciparum *prevalence (%)		*p*-value^a^
		
	Pre-intervention	Post-intervention	
Intervention (n = 68)	30.9	10.3	0.003
Control (n = 63)	9.5	15.9	0.285

^a^, Chi-square test

## Discussion

After the intervention, the malaria prevalence was significantly decreased in school children in the intervention school, although no decrease was observed in children in the control schools. Previous studies conducted in Ghana reported that falciparum prevalence was higher in the rainy season than in the dry season, and a little higher in the middle of the rainy season than at the beginning or end [21,22]. In the present study, pre- and post-intervention surveys were conducted at the end and middle of the rainy season, respectively. As is consistent with the trend reported from the previous studies, there was a slight increase in prevalence in the control schools at the post-intervention survey. Therefore, in the intervention school the effect of seasonal variation is unlikely to be the principal reason for the decrease. Although a marked difference in prevalence between intervention and control schools was observed at the baseline, intervention impacts can be considered as one of the main factors influencing prevalence reduction.

Previous studies conducted in sub-Saharan African countries including Ghana showed that people believed that not only mosquito bites, but also eating mango, drinking dirty water, and being exposed to hot sun were causes of malaria [17,19,20,23,24]. The same finding was observed in this study at pre-intervention. As Table 4 shows, 95.2% of the community adults correctly answered that mosquito bites can cause malaria. However, only a small proportion of the respondents disagreed with the incorrect statements that "heat from the sun", "eating mango", and "drinking dirty water" cause malaria. After the intervention, this knowledge was significantly improved in the intervention area. The improvement is important because a lack of understanding of the linkage between malaria and mosquito bites is associated with poor adherence to vector control interventions [20,25].

In this study, both community adults and school children showed significantly increased knowledge in the item "Paracetamol alone cannot cure malaria". In Ghana, most malaria cases have been managed at the household level [2]. However, during the fever episode, nearly 40% of children under 5 years old were not treated with any anti-malaria medicine [3], and in the absence of anti-malarials, paracetamol alone was commonly administered as treatment [18]. Knowledge improvement regarding treatment could be beneficial even to children. In Kenya, Geissler *et al *[26] reported that a considerable number of children self-treated their febrile illness without help from their caregivers.

In Ghana, most children under five years of age have yet to be protected by ITNs [3]. It has been emphasised that untreated conventional nets should be treated with insecticide to increase coverage of ITNs [3]. The observation survey found that 45% of the nets that respondents showed us were conventional nets. In response to the intervention, community adults who treated nets with insecticide increased from 21.5% to 50.0%, compared with the 25.3% to 30.5% in the control area. This fact suggests that the intervention was effective in increasing the coverage of ITNs.

Although the extra opportunity to treat their nets was provided to community people during the one-day campaign, providing an opportunity alone is unlikely to increase the net treatment rate. According to the local health authority, community people rarely participate in free net treatment services which health workers offer regularly in the study villages. Previous studies reported that barriers of insecticide-treatment were not only cost and access to treatment place, but also fear about insecticide, and poor linkage between malaria and ITNs [27-29]. Thus, community awareness raised by the children about the malaria likely had a substantial impact on increasing net treatment practices.

The results also showed that the frequencies of talking with children and guardians/neighbours about malaria unexpectedly decreased at the post-intervention survey. This trend was seen both in intervention and control groups. There is a possibility that community adults were busier at post-intervention period than pre-intervention period because of seasonally related changes to farming labour intensity. Unexpectedly, at the baseline, the frequency of talking between children and guardians/neighbours in intervention groups was much higher than those in control groups. This might be due to a higher burden of malaria, suggested by the higher prevalence at pre-intervention among school children in the intervention school.

This study has five major limitations. First, although conventional ITNs and long-lasting insecticide-treated nets (LLITNs) co-existed in the study site, no attempt was made to teach the respondents to recognize the two major differences to avoid confusion which might arise among study participants; conventional ITNs should be regularly treated and not be washed frequently. In contrast, LLITNs have no need of treatment until nets are washed many times and should be washed to activate insecticide agents. This limitation might be reflected in the result showing that knowledge "Necessity of ITN re-treatment" was not improved in the intervention area. Additionally, almost all of the community respondents reported that they washed their nets "very often" or "sometimes" in the past six months, although some of the nets must have been conventional ITNs. Second, although we used the local word "Asra" to define malaria, "Asra" does not necessarily correspond to "malaria" as defined by modern medicine. As shown in the previous study in Ghana, "Asra" was used interchangeably to define both malaria and fever [19]. Third, in data analysis, clustering of individuals within the same school was not taken into account, resulting in failure in addressing the cluster effects including class differences within the school. Fourth, results of the parasitological survey might be biased because of selection bias. Although no age and sex difference was observed between children who participated in the survey and those who did not, sex difference was observed in the children in the control schools. Finally, a randomised controlled design was not employed. However, no baseline difference was observed in demographic characteristics of the study participants and bed net related characteristics.

For the intervention, schoolteachers successfully adopted education activities using the PLA approach, such as role-playing, poetry recitals, slogan chanting, song composition and dramatization. These activities could be socially and culturally acceptable, because teachers themselves designed these activities. The results showed that most of the community adults were exposed to these activities. Moreover, participants in the intervention area were more likely to talk about malaria than those in the control area. Thus, the application of this strategy in other malaria endemic areas is recommended.

Scaling up school-based health education interventions should be easy if a well-established school health system is available. The study in Thailand utilized a school health system based on the Health Promoting School concept [14]. In recent years, WACIPAC has introduced the strategy for the setting up and establishment of school health management systems as a national programme in 10 West African countries. Moreover, WACIPAC has recommended the necessity of coordination among donors interested in school health. Partnership has been promoted between governments in target countries and donors for the establishment of school health management systems. Thus, school-based intervention has the potential to be scaled up on the basis of the systems in each country in West Africa.

## Conclusions

School-based malaria education intervention engaging school children as health messengers had a substantial impact not only on school children, but also on community adults in improving knowledge on cause and prevention and bed net impregnation practices. The improved knowledge and practices could be associated with the decrease in the malaria prevalence observed in the school children.

## Competing interests

The authors declare that they have no competing interests.

## Authors' contributions

IA and DN were the principal investigators and responsible for the whole process. JKA coordinated field implementation and contributed to the data collection. SH and JK contributed to field implementation, data analysis and manuscript drafting. MJ, TM, and TT contributed to reviewing the manuscript. KMB and DAB were involved in protocol development and contributed to study design development. All authors read and approved the final manuscript.

## Supplementary Material

Additional file 1 **Appendix: Teacher training programme**. The appendix shows detailed contents of the teacher training and teaching aids used in the training. Click here for file
