# Supplementary material for: School-based participatory health education for malaria control in Ghana: engaging children as health messengers
Source: Malar J. 2010 Apr 18;9:98. doi: 10.1186/1475-2875-9-98 (PMC2865503; doi:10.1186/1475-2875-9-98)
Supplement: Additional file 1 — Appendix: Teacher training programme. The appendix shows detailed contents of the teacher training and teaching aids used in the training. [file 1475-2875-9-98-S1.DOC]

**Appendix: Teacher training programme.**

| Day | Sessions | Contents | Teaching aids |
| --- | --- | --- | --- |
| Day 1 | Introduction to the training | - Explanation of the importance of malaria control at school - Explanation of the study objectives and purposes of the training - Feedback of the results obtained from pre-intervention surveys - Discussions | - Study proposal - Results of the questionnaire-based interview (Power Point slides) |
|  | Lecture on malaria | - Transmission mechanism - Mosquito biology (malaria vectors, life cycle, breeding and resting places, feeding habits) - *Plasmodium spp* biology (life cycle) - Signs and symptoms (common symptoms for uncomplicated malaria and critical danger signs) - Diagnosis and treatment (the importance of early diagnosis and prompt treatment, treatment regimen for uncomplicated malaria based on the National Policy, and drug resistance) - Prevention (use and maintenance of ITN, removal of mosquito breeding places in the community, and other preventive measures) | - Picture posters - Relevant pictures taken from the community in the observational survey (Power Point slides) - Mosquito larvae collected from the community - ITN |
|  | Lecture on PLA approach | - What is PLA? - Examples of PLA used for malaria control and other purposes. - Exercises in PLA | - Studies using the PLA approach for malaria control [1-3] - Literature describing various PLA based on the Health Promoting School concept [4] |
|  | Summary/ discussions |  |  |
| Day 2 | Action plan development | - Planning of PLA-based activities for the intervention - Review and fixing of activities on existing lesson time table used at the school - Assignment of responsibilities in implementing activities to teachers - Assessment of tools necessary for the implementation of activities | - Existing lesson time table used at the school |
|  | Closing ceremony | - Commitment from the school master |  |

**References**

1. Okabayashi H, Thongthien P, Singhasvanon P, Waikagul J, Looareesuwan S, Jimba M, Kano S, Kojima S, Takeuchi T, Kobayashi J, Tateno S: **Keys to success for a school-based malaria control program in primary schools in Thailand.** *Parasitol Int* 2006, **55:**121-126.

2. Onyango-Ouma W, Aagaard-Hansen J, Jensen BB: **The potential of schoolchildren as health change agents in rural western Kenya.** *Soc Sci Med* 2005, **61:**1711-1722.

3. Nonaka D, Kobayashi J, Jimba M, Vilaysouk B, Tsukamoto K, Kano S, Phommasack B, Singhasivanon P, Waikagul J, Tateno S, Takeuchi T: **Malaria education from school to community in Oudomxay province, Lao PDR.** *Parasitol Int* 2008, **57:**76-82.

4. Hawes H (Ed): *Health promotion in our schools.* London: Child-to-Child Trust; 1997.
